# Thermosensitive Gels Used to Improve Microneedle-Assisted Transdermal Delivery of Naltrexone

**DOI:** 10.3390/polym13060933

**Published:** 2021-03-18

**Authors:** Kevin V. Tobin, Jennifer Fiegel, Nicole K. Brogden

**Affiliations:** 1 Department of Pharmaceutical Sciences and Experimental Therapeutics, University of Iowa College of Pharmacy, Iowa City, IA 52242, USA; kevin-tobin@uiowa.edu; 2 Department of Chemical and Biochemical Engineering, University of Iowa College of Engineering, Iowa City, IA 52242, USA; jennifer-fiegel@uiowa.edu; 3 Department of Dermatology, University of Iowa Hospitals and Clinics, Iowa City, IA 52242, USA

**Keywords:** microneedle, poloxamers, transdermal drug delivery, naltrexone

## Abstract

Transdermal delivery of naltrexone (NTX) can be enhanced using microneedles, although micropores generated this way can reseal by 48 h in humans, which prevents further drug delivery from a formulation. Poloxamer 407 (P407) is a thermosensitive polymer that may extend microneedle-assisted NTX delivery time by creating an in situ gel depot in the skin. We characterized gelation temperature, drug release, and permeation of P407 gels containing 7% NTX-HCl. To investigate microneedle effects on NTX-HCl permeation, porcine skin was treated with microneedles (600 or 750 μm length), creating 50 or 100 micropores. The formulations were removed from the skin at 48 h to simulate the effect of micropores resealing in vivo, when drug delivery is blunted. Gelation temperature increased slightly with addition of NTX-HCl. In vitro NTX-HCl release from P407 formulations demonstrated first order release kinetics. Microneedle treatment enhanced NTX-HCl permeation both from aqueous solution controls and P407 gels. Steady-state flux was overall lower in the P407 conditions compared to the aqueous solution, though ratios of AUCs before and after gel removal demonstrate that P407 gels provide more sustained release even after gel removal. This may be beneficial for reducing the required application frequency of microneedles for ongoing treatment.

## 1. Introduction

Naltrexone hydrochloride (NTX-HCl) is a μ-opioid receptor antagonist used to treat alcohol and opioid dependence, and it is commercially available as oral tablets and an intramuscular depot injection. These preparations suffer from challenges such as extensive first-pass metabolism and injection site pain, respectively, and a more patient-friendly dosage form could improve the use of this medication in patients requiring chronic treatment. A transdermal product could offer advantages such as consistent plasma concentrations, ease of use, and increased patient satisfaction. However, NTX-HCl does not absorb well through the skin because of the physicochemical properties of the molecule. One method for improving delivery of NTX-HCl through the skin is through application of microneedles.

Solid microneedles (MNs) are small projections ranging from 100–1000 μm in length, made of materials such as hard polymers or stainless steel. When applied to the skin, solid MNs create aqueous micropores in the epidermis. A topically applied drug (in a patch or gel) can absorb through the micropores and reach the dermal blood supply for systemic absorption. This type of 2-step delivery approach in which solid MNs are used as a “pretreatment” has been successful for transdermal delivery of NTX-HCl in animals and humans [1,2,3], achieving similar steady state plasma drug concentrations as oral and intramuscular delivery.

When using solid MNs as a pretreatment to create micropores in the skin, the length of time that a drug can absorb through the skin is directly related to how long the micropores remain open at the skin surface. Previous studies in animals and humans have shown that the micropores close by ~48 h, which is likely related to the natural healing processes of the skin [4,5,6]. At that time further absorption of NTX-HCl from the formulation stops [1,7,8]. This means that a new MN application would need to be repeated every 2 days to maintain continuous transdermal NTX delivery, presenting potential challenges for patient compliance. In order to use MNs in a clinical setting for treatment of opioid and alcohol dependence, a novel formulation approach is needed so that MNs do not need to be reapplied so frequently.

Thermosensitive poloxamer gels could be a novel approach to reduce the frequency of MN applications and allow longer NTX-HCl delivery times from each MN treatment. Poloxamers in solution are liquid at lower temperatures and form gels as the temperature increases (this change occurs at a point known as the gelation temperature). When used in combination with MN pretreatment, a chilled liquid poloxamer solution containing NTX-HCl would quickly enter into the micropores. A slowly releasing gel would form as the poloxamer temperature increases in the skin, which would create an in situ gel depot in the micropores. This gel depot would continue to deliver NTX-HCl even after the micropores close at the skin surface.

Poloxamer 407 (P407) has been used in conjunction with MN pretreatment to provide sustained drug release of other drugs in vitro [9,10,11], but the effects of MN length and number on drug absorption from poloxamers have not been investigated. Further, this approach has not been studied with NTX-HCl. The objectives of the present study were to (1) characterize the gelation temperature of P407 formulations loaded with NTX-HCl, and (2) investigate effects of MN length and number of micropores on NTX-HCl permeation from P407 gels.

## 2. Materials and Methods

### 2.1. Materials

NTX-HCl was obtained from Mallinckrodt Pharmaceuticals (Webster Groves, MO, USA). P407 was obtained from Anatrace (Maumee, OH, USA). HEPES and sodium bicarbonate were obtained from Research Products International (Mt. Prospect, IL, USA). Sodium hydroxide (1N), o-phosphoric acid, and methanol Optima^®^ were obtained from Fisher Chemical (Lenexa, KS, USA). Octanesulfonate sodium, Hank’s balanced salts, HPLC grade water, and HPLC grade acetonitrile were obtained from Sigma Aldrich (St. Louis, MO, USA). Snakeskin^®^ cellulose acetate membrane (10,000 MWCO) was obtained from Thermo Scientific (Rockford, IL, USA). Solid stainless steel microneedle arrays of 600 μm or 750 μm length (200 μm width, 75 μm thickness, 1.3 mm inter-needle spacing for both) consisting of 50 projections bent 90° out of plane were purchased from Tech Etch (Plymouth, MA, USA).

### 2.2. Preparation of NTX-HCl Poloxamer Gels

The required mass of P407 to make 17%, 18%, 19% and 20% *w*/*w* solutions was added to deionized water and rotated overnight at 4 °C to fully dissolve and create clear, viscous solutions. The appropriate mass of NTX-HCl to make 7.0% *w*/*v* NTX-HCl formulations was added to the P407 solutions and rotated overnight at ambient temperature (~21 °C) to fully dissolve and create clear pale yellow, viscous solutions. This concentration of NTX-HCl was selected based on results of initial solubility studies (described below). Blank P407 formulations were stored at 4 °C and P407 solutions loaded with NTX-HCl were stored at ambient temperature and protected from light to avoid significant precipitation, NTX-HCl degradation, or bacterial growth; samples were stored for no more than one week.

### 2.3. Solubility Studies

The solubility of NTX-HCl in deionized water and P407 solutions was measured at 20 °C to determine the formulation concentration used in the present studies. This temperature was selected because it was just below measured ambient temperature (~21 °C), which would allow the maximum amount of NTX-HCl to be dissolved without gelation at ambient conditions. The solubility of NTX-HCl in water at 25 °C was also measured as a control to compare with values reported in the literature. Excess NTX-HCl was added to 1 mL of each P407 solution and samples were gently shaken overnight in a water bath set to the appropriate temperature. The next day samples were spun down in a centrifuge at 17.0× *g* for 10 min. The supernatant was diluted with deionized water before the concentration was measured using high-performance liquid chromatography (HPLC).

### 2.4. Gelation Characteristics of P407 Solutions

The gelation temperature of P407 formulations with and without NTX-HCl was determined using a stir bar method that has been described previously [12]. A cooled 20 mL scintillation vial containing the P407 formulation and a magnetic stir bar was placed on a hot plate that slowly heated the formulation with constant stirring. A thermometer was submerged in the formulation just above the stir bar. When the gel formation was sufficient to stop the stir bar from rotating freely, the temperature on the thermometer was recorded. Studies were performed in triplicate.

A rheological method was used as a complementary approach to verify the gelation temperature of P407 formulations. An ARES-G2 rheometer (TA Instruments, New Castle, DE, USA) was used, outfitted with a Peltier plate and 50 mm diameter parallel plates equipped with a solvent trap. The gap between the plates was set to 0.5 mm for all experiments. Amplitude sweeps from 0.01 to 100% strain were performed at 37 °C with a 1 Hz frequency, and frequency sweeps from 0.1 to 100 Hz were performed at 37 °C with 0.2% strain for each formulation to determine the linear viscoelastic region (LVR) of the gels. The LVR provided the appropriate strain and frequency parameters to avoid structural breakdown of the gel network during oscillatory tests to determine the gelation temperature. The Winter–Chambon method was used to verify the gelation temperature findings from the stir bar method. According to this method, the point of gelation occurs when the loss tangent is independent of frequency [13]. Frequency sweeps were performed from 0.1 to 100 Hz, increasing logarithmically, at constant temperature with stepwise temperature increase of 0.1 °C and 1 min equilibration after each temperature increase. The temperature at which the loss tangent curves cross for different frequencies was considered the gelation temperature. Studies were performed in triplicate.

### 2.5. In Vitro Release Studies

Franz diffusion cells with a 5 mL receiver compartment and 0.64 cm^2^ diffusion area (Permegear, Hellertown, PA, USA) were used to quantify NTX-HCl release in vitro. The receiver compartment was filled with filtered receiver solution containing 0.592% *w*/*w* HEPES buffer (pH 7.4) with Hank’s balanced salts and 0.035% *w*/*w* sodium bicarbonate, pre-warmed to 37 °C. Cellulose acetate dialysis membrane (10,000 MWCO) was used as the membrane between the donor and receiver compartments. Two hundred microliters of donor formulation containing 7.0% NTX-HCl was applied to each cell and 0.3 mL of receiver solution was collected at 5, 15, 30 and 45 min followed by 1, 1.5, 2, 2.5, 3, 4, 5, 6, 12, 18 and 24 h. Fresh, pre-warmed buffer was added back to the receiver compartment after each time point to maintain sink conditions. Samples were stored at 4 °C until diluted and analyzed via HPLC.

### 2.6. Skin Preparation

Dorsal skin from Yucatan miniature pigs was obtained from Sinclair Bio Resources, LLC (Auxvasse, MO, USA) and stored at −80 °C until use. For permeation studies, skin samples were allowed to thaw at room temperature and were used for an experiment the same day as thawing. Skin samples were dermatomed to 1 mm thickness (Nouvag, Model TCM3000BL, Goldach, Switzerland) and cut to the appropriate size to cover the entire diffusion area of the in-line diffusion cells (described below). Select skin samples were pretreated with MN arrays consisting of 50 stainless steel MNs of 600 or 750 μm length. To create 50 micropores, the arrays were applied to the skin samples using gentle thumb pressure for 15 s. For the 100 micropore condition the array was removed after the first application, rotated 45°, and then applied to the skin a second time. If damage to the MNs was observed (broken needles, altered angle of projection), a new array was used. Skin not pretreated with MNs served as controls.

### 2.7. In Vitro Permeation Studies

**Figure 1:**
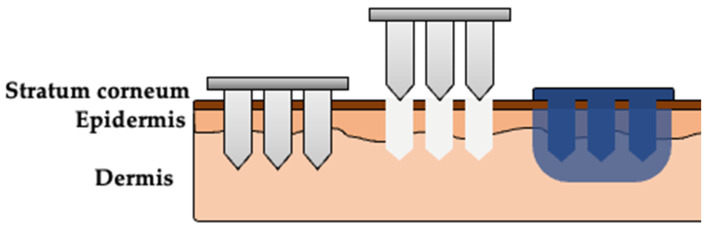
Visual representation of the multi-step skin treatment approach used in the in vitro permeation studies. Solid microneedles (MNs) are used to create micropores in the skin. A formulation (aqueous solution or P407 gel) is applied over the area treated with MNs, enters the micropores, and delivers drug into the skin. The poloxamer solution will form an in situ gel depot after reaching skin temperature.

In-line diffusion cells with 1.77 cm^2^ diffusion area (Permegear, Hellertown, PA, USA) were used to quantify NTX-HCl permeation through porcine skin in vitro. Receiver solution (same as described above for release studies) was warmed to 37 °C and pumped at a flow rate of 1.5 mL/h to maintain sink conditions. The prepared skin samples were mounted in the diffusion cells, warmed to 32 °C using a manifold, and allowed to equilibrate for ~30 min before beginning the experiment. Five hundred microliters of donor formulation containing 7.0% NTX-HCl was applied to the diffusion area to begin the study, and the diffusion cells were occluded for the duration of the experiment after dose application. Receiver solution was collected in 3 h increments for 72 h. Donor formulation was carefully removed at 48 h using a cotton swab or Kimwipe^®^. The purpose of this was to mimic the closure of micropores in vivo that blunts further drug absorption from the formulation above the stratum corneum, and to quantify delivery from the drug depot in the skin that formed during the first 48 h. After collection, the samples were stored at 4 °C until being filtered and analyzed using HPLC. All conditions were performed with 3 to 6 replicates. A visual depiction of the skin treatment process is shown in Figure 1.

### 2.8. HPLC Method for NTX-HCl Detection

The concentration of NTX-HCl in samples was determined using reverse-phase HPLC on a Shimadzu Prominence *i*-Series LC-2030 Plus system with UV detector (Shimadzu, Torrance, CA, USA), outfitted with a Phenomenex^®^ (5 μm particle size, 100 Å pore size, 150 mm length, 4.6 mm inner diameter) C-18 column (Phenomenex, Torrance, CA, USA). NTX-HCl absorption was detected at 280 nm. The mobile phase consisted of 25:75 acetonitrile:HPLC buffer (0.065% *w*/*v* octanesulfonate sodium and 0.13% *v/v* o-phosphoric acid in water) at a flow rate of 1 mL/min and a run time of 8 min. Samples that were obtained from skin permeation studies were measured with the mobile phase shifted to 18:82 acetonitrile:HPLC buffer at a flow rate of 1 mL/min and run time of 12 min to separate the NTX-HCl peak from peaks generated by skin components. A 10 μL injection volume was used for all samples.

### 2.9. Data Analysis

For the Winter–Chambon gelation temperature determination method, frequencies below 1.0 Hz and above 50.12 Hz were not included in the data analysis because they were inconsistent with trends found at frequencies between 1.0 and 50.12 Hz, suggesting that frequencies outside of this range do not fit the Winter–Chambon equation for our system. In vitro NTX-HCl release from the P407 formulations was fitted to various release models to obtain kinetic release constants. Zero-order, first-order, and Higuchi kinetic release models are frequently used to describe drug release from P407 formulations. The Korsmeyer–Peppas release equation is used to further characterize the mechanism of drug release from polymeric systems.

Zero-order release equation:

(1) $$Q_{t}=\;K_{0}\cdot t$$

where $Q_{t}$ is the cumulative mass of drug released at time *t* and *K*_0_ is the zero-order rate constant with units of mg/h.

First-order release equation:

(2) $$\log\left(Q_{r}\right)=\frac{-K_{1}t}{2.303}$$

where *Q_r_* is the cumulative mass of drug remaining in the donor compartment and *K*_1_ is the first-order rate constant with units of h^−1^.

Higuchi release equation:

(3) $$Q_{t}=\;\;K_{H}\sqrt{t}$$

where *K_H_* is the Higuchi rate constant with units of mg/h^1/2^.

Korsmeyer–Peppas release equation:

(4) $$\frac{Q_{t}}{Q_{\infty }}=\;K_{K-P}\cdot t^{-n}$$

where $Q_{t}/Q_{\infty }$ is the fraction of of drug released at time *t*, *K_K−P_* is the Korsmeyer–Peppas rate constant with units h^−n^, and *n* is the drug release exponent (related to the drug release mechanism) [14]. Data were fitted for the first 60% of drug release to limit the influence of donor drug depletion, and the rate constant from the model with the best fit was used to compare the release of different formulations.

Any permeation samples deemed to be outliers (measured concentrations more than one magnitude greater than samples collected immediately before and after) were removed and replaced using linear interpolation between the sample immediately before and after (n = 8 individual samples fit these criteria). The cumulative mass of NTX-HCl permeated was plotted versus time and the apparent steady state flux (J_ss_) was calculated from the slope of the linear portion of the curve. The flux enhancement between MN conditions was estimated by taking the ratio of steady state flux through MN treated skin over the steady state flux through intact skin. The area under the curve (AUC) before and after formulation removal were calculated using the linear trapezoidal rule. All data were performed in triplicate and statistical analysis was conducted using GraphPad Prism 8 (GraphPad Software, Inc., La Jolla, CA, USA). One-way ANOVA with Tukey’s multiple comparison was used, and data are reported as mean ± SD. A *p*-value of <0.05 was considered statistically significant.

## 3. Results

### 3.1. Solubility Studies

The solubility of NTX-HCl in various concentrations of P407 was measured and compared to aqueous solution solubility controls. The solubility of NTX-HCl in aqueous solution at 25 °C was 102.9 ± 0.64 mg/mL, which is comparable to the solubility (100 mg/mL) recorded in literature [15]. The solubility in aqueous solution at 25 °C was significantly higher than all formulations tested at 20 °C in aqueous solution or P407 (*p* < 0.0001). NTX-HCl solubility in 17, 18, 19 and 20% *w*/*w* P407 formulations at 20 °C was 77.75 ± 1.21, 79.12 ± 0.78, 78.94 ± 1.08 and 74.04 ± 1.49 mg/mL, respectively; these were not significantly different from each other or from aqueous solution at 20 °C (82.49 ± 1.06 mg/mL); *p* > 0.05. A concentration of 70 mg/mL NTX-HCl (7.0% *w*/*v* NTX-HCl) was below the solubility limit for every formulation and was selected for ongoing studies to avoid potential challenges (such as drug precipitation) that may arise when using oversaturated solutions.

### 3.2. Gelation Characteristics of P407 Solutions

Gelation temperatures of P407 formulations with and without NTX-HCl were experimentally determined using the stir bar method, and the gelation temperature decreased with increasing P407 concentration. The gelation temperatures were measured as 27.93 ± 0.55, 25.30 ± 0.17, 23.73 ± 0.06 and 22.37 ± 0.15 °C for 17%, 18%, 19% and 20% *w/w* P407 formulations without drug, respectively. The addition of 7.0% NTX-HCl increased the gelation temperature by 2.82 ± 0.46 °C on average for all P407 concentrations.

**Figure 2:**
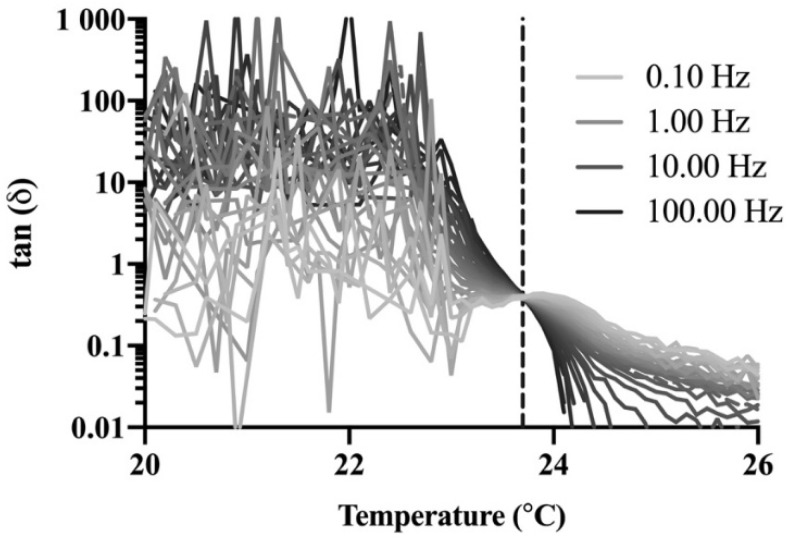
Representative plot (n = 1) of the loss tangent as a function of temperature at frequencies between 0.10 and 100 Hz for 20% P407 containing 7.0% naltrexone hydrochloride (NTX-HCl). The vertical dashed line depicts the temperature (23.7 °C) at which the loss tangent is independent of frequency. Studies were performed with n = 3 and the curve shape was similar for all formulations and P407 concentrations, so n = 1 is provided here for clarity in presentation.

**Figure 3:**
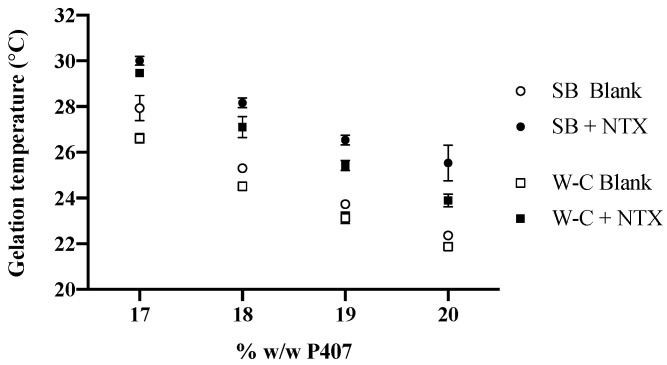
Comparison of experimentally determined gelation temperature for P407 formulations with (filled symbols) and without (open symbols) 7.0% NTX-HCl, using the stir bar (SB) and Winter–Chambon (W–C) methods. n = 3, data presented as mean ± SD (some error bars are too small to be visible on the graph).

A rheological technique was used as a complementary method to confirm the gelation temperatures obtained with the stir bar method for P407 formulations. Amplitude and frequency sweeps were performed for P407 formulations with and without NTX-HCl to determine the LVR for each gel. All amplitude and frequency sweeps showed that the LVR was maintained until 0.3% strain at a 1 Hz frequency for a 0.5 mm gap (data not shown). The LVR was maintained for the range of 0.1 to 100 Hz at a 0.2% strain and 0.5 mm gap (data not shown). A strain of 0.2% and a frequency of 1 Hz was used for ongoing studies in order to stay within the LVR during oscillatory tests. Frequency sweeps were performed to determine the gelation temperature using the Winter–Chambon method and a representative plot of the loss tangent, tan(δ), over temperature for different frequencies is shown in Figure 2. There is a cross-over point at which the loss tangent is independent of frequency, which was recorded as the gelation temperature. All formulations showed the same general plot shape with different temperatures where the frequency was independent. The resulting phase diagrams for P407 formulations with and without 7.0% NTX-HCl comparing the gelation temperature determined with the stir bar vs. Winter–Chambon method are shown in Figure 3. The Winter–Chambon method produced slightly lower (by 0.94 ± 0.51 °C) gelation temperatures compared to the stir bar method.

### 3.3. In Vitro Release Studies

**Figure 4:**
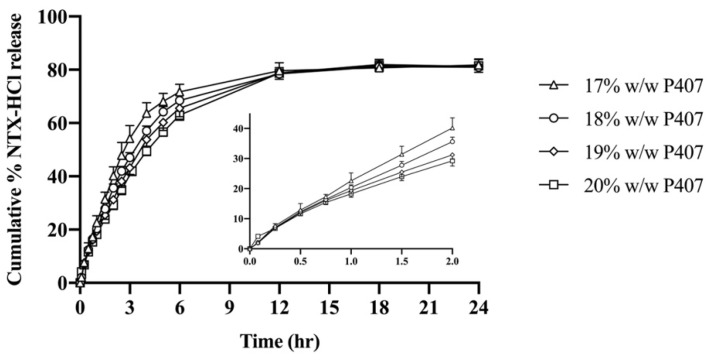
Cumulative release of NTX-HCl from P407 gels through cellulose acetate membrane. All donor formulations contained 7.0% NTX-HCl (n = 4, data presented as mean ± SD). Graph insert shows early time points.

NTX-HCl release from P407 formulations was quantified in vitro. The cumulative release of NTX-HCl over 24 h is shown in Figure 4. Over 60% of the NTX-HCl initially in the donor compartment was released by 3–6 h for all formulations, and 77.65 to 84.80% was released by 24 h. The data were fitted to zero-order, first-order, Higuchi, and Korsmeyer–Peppas release kinetics models for the first 60% of release (to avoid significant influence of donor drug depletion). The parameters for all four kinetic models are displayed in Table 1, showing that the data best fit first-order release (r^2^ > 0.996). The first order release rate constant for NTX-HCl from 20% P407 (0.17 ± 0.01 h^−1^) was lower than from 17% P407 (0.26 ± 0.03 h^−1^), 18% P407 (0.23 ± 0.01 h^−1^) and 19% P407 (0.19 ± 0.01 h^−1^). Therefore, the 20% P407 formulation was selected for further testing because these data suggest that it would be most suitable for prolonging the release of NTX-HCl from the in situ depot in MN treated skin.

### 3.4. In Vitro Permeation Studies

**Figure 5:**
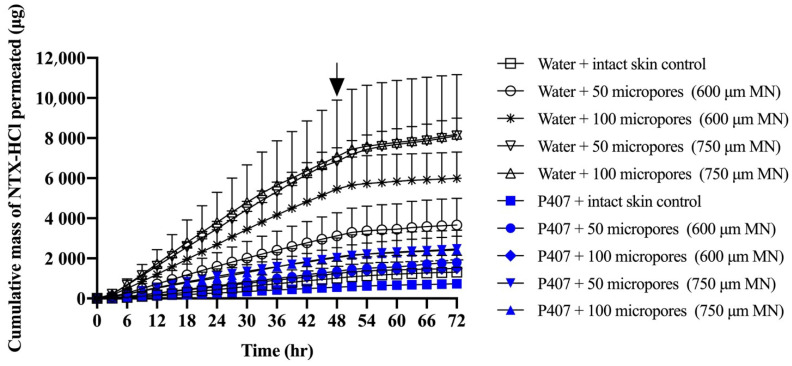
Cumulative mass of NTX-HCl permeated through intact or MN treated porcine skin from aqueous solution or 20% P407 gels; all formulations contained 7% NTX-HCl. Arrays of MNs, 600 or 750 μm length, were applied to generate 50 or 100 micropores. Arrow at 48 h denotes when formulations were removed from the skin surface. Data presented as mean ± SD (n = 3–6). MN = microneedles.

**Figure 6:**
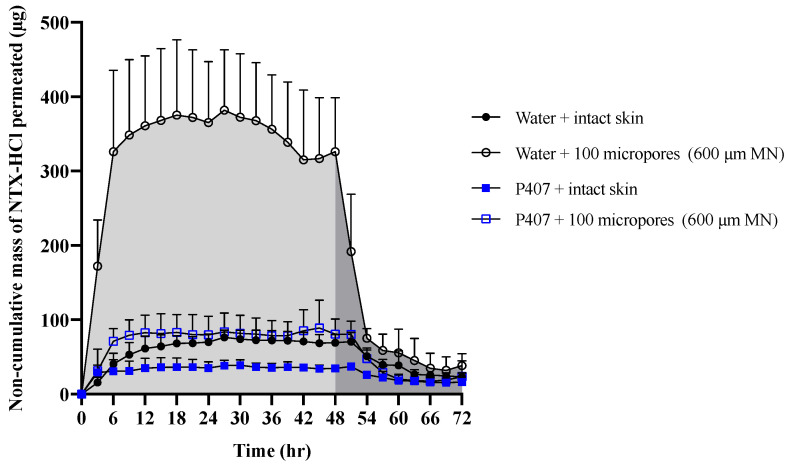
Representative curve of non-cumulative mass of NTX-HCl permeated through intact and MN-treated porcine skin from aqueous solution or 20% P407 gels over 72 h; all formulations contained 7% NTX-HCl. Light shaded areas represent AUC_0–48 h_ (before formulation removal) and dark shaded areas represent AUC_48–72 h_ (after formulation removal). Data presented as mean ± SD (n = 3–6). MN = microneedles.

The cumulative and non-cumulative permeation of NTX-HCl through MN treated porcine skin from aqueous solution and 20% P407 gels was quantified (Figure 5 and Figure 6). The cumulative mass permeated from 9 to 24 h showed sufficient linearity (r^2^ > 0.995) for every condition and was used to calculate steady state flux (Table 2). Intact skin controls produced steady state flux values of 12.56 ± 2.25 and 6.77 ± 2.17 μg/cm^2^/h for aqueous solution and P407 gels, respectively. The steady state flux from aqueous solution increased significantly compared to intact skin when 600 μm length MNs were used to generated 100 micropores (69.58 ± 17.69 μg/cm^2^/h) and when 750 μm length MNs were used to generate 50 (87.55 ± 10.24 μg/cm^2^/h) or 100 micropores (98.05 ± 38.15 μg/cm^2^/h) (*p* < 0.05). The steady state flux from P407 gels was significantly different between intact skin and skin treated with 750 μm MNs used to generate 50 (26.94 ± 6.31 μg/cm^2^/h) and 100 micropores (24.58 ± 10.39 μg/cm^2^/h); *p* < 0.05. The formulations were removed at 48 h in order to simulate the blunting of drug absorption from the donor formulation upon micropore closure in vivo. After removal of donor formulation at 48 h, a leveling off was observed in the cumulative permeation curve by 51 h.

**Figure 7:**
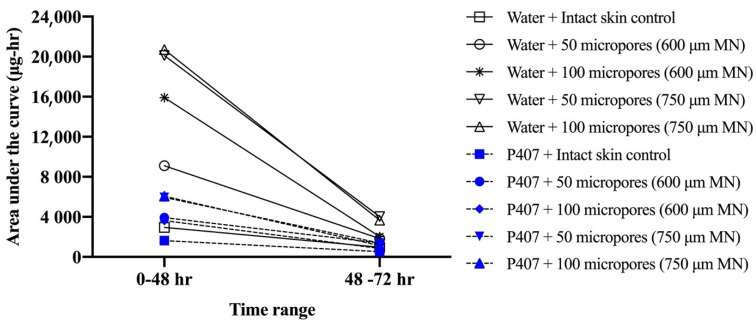
Comparison of mean area under curve (AUC) values before (0–48 h) and after (48–72 h) formulation removal for mass of NTX-HCl permeated through intact or MN-treated porcine skin from aqueous solution or 20% *w/w* P407 gels; all formulations contained 7% NTX-HCl (n = 3–6). Error bars omitted for clarity.

Figure 6 shows representative curves for non-cumulative mass of NTX-HCl permeated through intact or MN treated skin for the 600 µm, 100 micropores condition. The non-cumulative plot was used to calculate the AUC from 0 to 48 h and from 48 to 72 h separately to estimate the total in vitro drug exposure before and after formulation removal. The difference between the AUC_0–48 h_ and AUC_48–72 h_ was compared in Figure 7, showing that the change is more drastic for water than for P407 gels on skin treated with MN.

## 4. Discussion

Alcohol and opioid dependence are disorders that require lifelong treatment which calls for regimens that are easy to adhere to, ensuring the lowest chance of relapse. Transdermal delivery of NTX-HCl would be a patient-friendly dosage form, and MNs can allow enough NTX-HCl to absorb through the skin to reach appropriate clinical plasma concentrations. A transdermal product that could extend the time between required MN applications would be of great clinical benefit, and thermosensitive poloxamers are well suited for this approach. The present studies were used to characterize the gelation properties of P407 formulations loaded with NTX-HCl and investigate the effect of MN length and micropore number on NTX-HCl permeation from P407 gels.

### 4.1. Effect of NTX-HCl on P407 Gelation Characteristics

Poloxamers are non-ionic triblock amphiphilic thermosensitive copolymers with hydrophilic ethylene oxide tails and a hydrophobic propylene oxide middle. It is generally accepted that when P407 is cold it remains as monomers in solution, but as it heats up the hydrophobic propylene oxide centers dehydrate. This increases the entropy of the system by decreasing water structuring [16,17]. Micelles form in the solution, and when these reach a certain concentration, rigidity and viscosity increase. This ultimately forms a gel [17,18,19]. The stir bar technique allowed for the gelation temperature of each formulation to be measured with a physical method that has low variability, and the results are comparable with previous reports for P407 formulations without drug [12]. Because additives such as salts and drugs have been shown to affect the gelation temperature [20,21,22,23], the P407 formulations loaded with 7.0% NTX-HCl were also evaluated; each P407 formulation demonstrated an increase in gelation temperature upon addition of the drug. The addition of NTX-HCl likely increases the gelation temperature due to NTX-HCl disrupting the hydration sphere around the hydrophobic portion of the P407. This would stabilize the system by decreasing its overall water structuring, reducing the need for entropically driven micellization [21]. Rheological studies were used as a complementary technique and verified the gelation temperatures with results that were within 2 °C of those obtained with the stir bar method (with and without NTX-HCl).

The gelation temperature is important for the proposed topical administration because if the gelation temperature is higher than skin temperature it will not form a gel after administration and would not provide the benefits of controlled release. Ji et al. showed that the temperature of human upper arm skin, a common site for transdermal patches, varied between 31 and 32 °C for a patient in an ambient condition of 20 °C [24]. All presently tested formulations required temperatures below 32 °C for gelation to occur. This suggests that these formulations will form a gel when in contact with the skin and thus are suitable for the proposed application.

### 4.2. NTX-HCl Release from P407 Gels In Vitro

Drug permeation can be prolonged from a P407 gel depot compared to solution [23,25,26]. Drug release from poloxamer gels can be affected by many factors including drug properties (such as the lipophilicity of the drug) and formulation properties (poloxamer concentration, formulation pH, gel erosion, and additives) [23,26,27]. In the present work, drug release was quantified with the goal of selecting a formulation that can provide prolonged delivery of NTX-HCl through MN treated skin. Consistent with literature [27], only 80% of the NTX-HCl was released from the P407 formulation, after which the cumulative release reaches a plateau. This could be due to NTX being preferentially retained in the P407 formulation over aqueous buffer.

In these studies, we compared four kinetic release models to describe the mechanism of NTX-HCl release from P407 gels; Table 1. We chose this approach because, based on previous literature, drug release from poloxamer gels can be fitted to several different models. The fitting to different models likely occurs because properties of both the drug and the gel formulation will affect the drug release, thereby changing the model that best describes the release. For example, the release of morphine, diclofenac sodium, and pilocarpine from P407 gels has been reported to follow zero-order release; in contrast, etoposide gels have been reported to follow first-order release, while benzoic acid, vancomycin and lidocaine gels follow Higuchi release [22,23,25,26,28,29,30,31]. Additionally, drug release data from poloxamer gels have been fitted to Korsmeyer–Peppas kinetic release models in attempts to further understand the release mechanisms [30,31].

Our data best fit a first-order kinetic release model, which describes a release rate that changes proportionally to the concentration of drug in the donor formulation (i.e., concentration of drug in the gel). This is often the model that describes release from drug either in solution or in matrix diffusion-controlled release. NTX is positively charged at the formulation pH (6.07 ± 0.29) which increases the hydrophilicity of the molecules. NTX molecules are likely to be outside of the micelles in the P407 network because the inner core is hydrophobic. NTX-HCl release may fit first order release kinetics best because the NTX-HCl molecules would need to diffuse through the aqueous channels between micelles to escape the formulation. When the data were fit to a Korsmeyer–Peppas model to further understand the dissolution mechanism from the polymer matrix, the release exponent (n) was between 0.5 and 1. This suggests that a non-Fickian transport mechanism is occurring, suggesting that diffusion and swelling govern the release of NTX-HCl [32]. These data provide additional support for the idea that P407 gels progressively slow the release rate of drugs in increasing P407 concentration, likely due to increased viscosity which slows the diffusivity of drugs [22,27].

### 4.3. Effects of MN Length and Number on Permeation In Vitro

NTX-HCl steady state flux through MN-treated skin is significantly affected by both the formulation composition and MN characteristics (length and number of MNs) [33,34]. Separately or combined, these factors can result in differences in flux, lag time, and time to steady state. Longer and/or increased number of MNs typically results in a longer time for micropore closure [4,35] but may also affect the level of permeation enhancement. Here we studied MNs of 600 and 750 μm length. Many previous permeation studies with NTX-HCl have been performed with MNs of 750 μm length. In the current study the steady state flux values from aqueous solutions applied to MN treated skin were higher than previously reported with similar formulations. However, one major difference is that the previous work was mostly done with gels containing propylene glycol in concentrations ranging from 10–75% [8,33,36]. Thus, the differences in flux that we observed are not unexpected given inter-study differences in skin thickness, skin types, diffusion area, and differences in formulation viscosity. NTX-HCl permeation from aqueous solution followed the trend that the 600 µm MNs were beneficial for enhancing flux, but flux did not significantly increase when going up to 750 µm MNs (*p* > 0.05); Table 2.

For the aqueous solution, flux enhancement ratios of 3.34 ± 1.32 and 5.54 ± 1.41 were achieved for the 600 µm MN condition with 50 and 100 micropores, respectively. Further enhancement was seen with the 750 µm MNs when 50 micropores were created, though the enhancement reached a threshold after which the increased number of micropores or MN length did not provide additional, proportional benefit. The enhancement ratio for the 20% *w*/*w* P407 gels followed a somewhat similar trend: 2.62 ± 0.81 and 2.27 ± 0.70 for the 600 µm MN condition with 50 and 100 micropores, respectively. This enhancement increased again when the longer 750 µm MNs were used to create 50 micropores, but beyond that no further enhancement was observed. Similar trends have been reported for other drugs. Gao et al. showed that cumulative permeation of naloxone was significantly higher using 700 μm compared to 500 μm length MNs [37]. Yan et al. determined that increasing MN length above 600 μm significantly enhanced flux of acyclovir compared to MN lengths less than 600 μm, though there was minimal difference between 600 and 750 μm lengths [34]. MN treatments with shorter projections and lower micropore numbers have the additional benefit that they may be more readily accepted by patients, because decreased length and number of MNs is reported to produce less discomfort [4]. An additional interesting finding in the present work was that the P407 gels showed less overall variability in NTX-HCl permeation compared to the aqueous solution. Reducing the variability in drug permeation will be especially important because in vivo delivery comes with added variability of micropore closure rates that are seen in different patient populations [35,38].

### 4.4. Effects of Pore Closure on NTX Permeation In Vitro

In order for NTX to be therapeutically effective *in vivo*, a plasma concentration of 2 ng/mL is required for sufficient opioid receptor blockade [39]. The first pharmacokinetic study in humans that used the MN pretreatment approach (also known as the “poke/press and patch” approach) used NTX-HCl as a model compound, achieving the 2 ng/mL plasma concentration benchmark [2]. Pharmacokinetic sampling confirmed relatively consistent plasma concentrations for 48 h, after which the NTX concentration began to decline. This has been attributed to closure/re-sealing of the micropores in vivo, which prevents further drug absorption from the gel formulation (though some absorption would still continue from any drug that formed a depot in the skin). Due to the in vitro nature of our current study, we were not able to simulate micropore closure. Instead, we tried to mimic the effect of micropore resealing by removing the formulation at 48 h. The rate of drug permeation was slowed after formulation removal for both formulations (aqueous vs. P407) and all skin conditions (intact vs. MN treated). This slowed rate of permeation can be observed on the cumulative permeation plot (Figure 5), in which the slope changes after 48 h. The slowed permeation rate can also be seen in the sudden drop on the noncumulative permeation curve (Figure 6) after 48 h.

After formulation removal from the skin surface, some drug permeation continues because of a drug depot that forms within the skin. This drug depot then allows continued drug absorption even after the formulation is not present at the skin surface. The rate of drug elimination from the skin after formulation removal was faster for aqueous solution than for P407 formulations, as shown by the drastically different slopes from 48–54 h in the noncumulative permeation plot (Figure 6). These data suggest that P407 is continuing to deliver drug from inside the micropores or skin after formulation removal, while aqueous solution delivery is disrupted when the formulation is removed from the skin.

**Figure 8:**
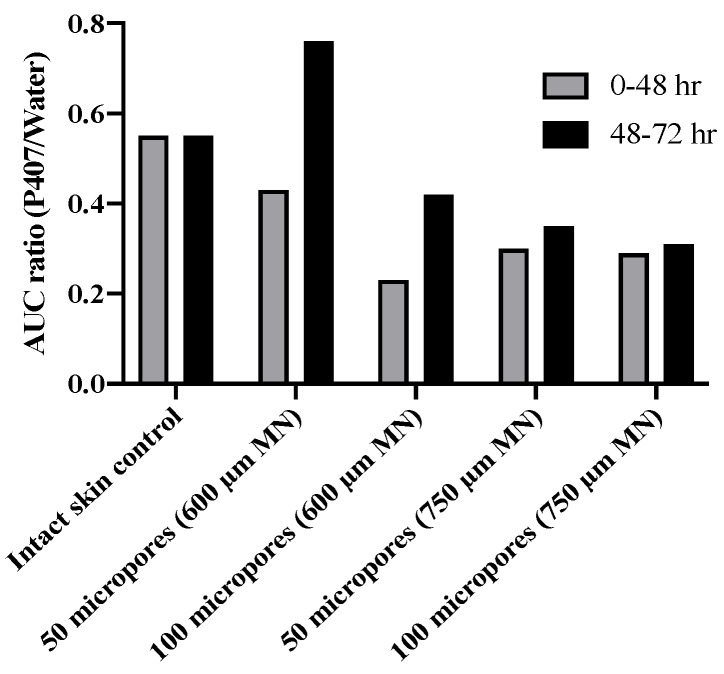
Ratio of mean AUC (20% P407/aqueous solution) of NTX-HCl permeation through intact or MN treated porcine skin for 0–48 h or 48–72 h; all formulations contained 7.0% NTX-HCl. No error bars are shown because ratios were calculated from two means.

From our results we were able to compare differences in drug “exposure” (how much drug permeates) before and after gel removal from the skin. This exposure can be quantified by the AUC, which we calculated for both 0–48 and 48–72 h timeframes, corresponding to before and after formulation removal, respectively. We also calculated a ratio of P407/aqueous solution AUCs. A ratio of <1 for all conditions suggests that P407 provides a more sustained and controlled delivery of NTX-HCl compared to the aqueous solution, even after formulation removal. The AUC ratios (Figure 8) were all similar except for 600 μm length MN conditions where the ratio is almost doubled, likely due to the rapid drop off in NTX-HCl permeation through the skin from aqueous solution. Our results showed that removing the formulation led to a more drastic decrease in in vitro drug exposure for aqueous solution compared to P407 gels. Collectively our data (enhancement ratios, flux values, and AUC ratios) suggest that 600 µm MNs may be an optimal length for NTX-HCl delivery from P407 gels.

### 4.5. Limitations and Future Directions

One of the limitations of the present in vitro permeation studies is the lack of a physiological healing response. Although the removal of formulation at 48 h was used to simulate cessation of drug absorption from formulation above the stratum corneum, this does not truly represent what occurs in vivo. Although drug absorption is blunted around 48 h due to micropore closure, the micropores all close at different rates leading to a gradual resealing until full barrier recovery [4,5]. To learn how the present formulations perform in a model with healing skin, studying NTX-HCl permeation in vivo in animals and humans will be necessary. The P407 formulations that we studied here contained no additional excipients to enhance the permeation or increase the concentration of NTX-HCl. If the concentration of NTX-HCl was increased with the use of cosolvents or buffers, then permeation would likely be synergistically improved because drug concentration directly affects the rate of diffusion. Chemical permeation enhancers could also be added to the formulation to improve drug absorption. Because additives can change the gelation characteristics of P407 formulations, additional testing will be necessary to characterize the performance of the new formulations. In ongoing work with permeation enhancers, it will also be necessary to perform studies using techniques such as FT-IR and NMR to characterize the chemical interaction(s) occurring within the gels.

## 5. Conclusions

The present studies focused on assessing permeation of NTX-HCl from P407 gels through MN treated skin in vitro. Gelation temperatures of P407 formulations were determined and verified, and the formulations formed gels before reaching skin temperature. In vitro permeation studies showed that P407 can provide sustained permeation of NTX-HCl through MN treated skin with lower variability than aqueous solution.

## Figures and Tables

**Table 1:** Release kinetic model parameters for P407 formulations containing 7.0% NTX-HCl (n = 4). Kinetic rate constants (K) are presented as mean ± SD; drug release exponent (n) and r^2^ values presented as mean.

P407 Concentration (% *w*/*w*)	Model Parameter	Zero Order	First Order	Higuchi	Korsmeyer–Peppas
17%	r^2^	0.992	0.998	0.972	0.991
	K	2.66 ± 0.35 mg/h	0.26 ± 0.03 h^−1^	3.73 ± 0.17 mg/h^1/2^	0.22 ± 0.02 h^−n^
	n				0.88
18%	r^2^	0.985	0.999	0.979	0.991
	K	2.21 ± 0.07 mg/h	0.23 ± 0.01 h^−1^	3.50 ± 0.11 mg/h^1/2^	0.20 ± 0.01 h^−n^
	n				0.85
19%	r^2^	0.986	0.996	0.980	0.985
	K	2.03 ± 0.10 mg/h	0.19 ± 0.01 h^−1^	3.21 ± 0.16 mg/h^1/2^	0.19 ± 0.01 h^−n^
	n				0.84
20%	r^2^	0.981	0.993	0.984	0.992
	K	1.85 ± 0.08 mg/h	0.17 ± 0.01 h^−1^	2.95 ± 0.13 mg/h^1/2^	0.19 ± 0.01 h^−n^
	n				0.65

**Table 2:** Steady state flux of NTX-HCl permeated through intact or MN-treated porcine skin from aqueous solution or 20% P407 gels over 9 to 24 h. All formulations contained 7% NTX-HCl. Data presented as mean ± SD (n = 3–6). MN = microneedles.

Formulation	Intact Skin (Control) (μg/cm^2^/h)	600 μm MN		750 μm MN	
		50 Micropores (μg/cm^2^/h)	100 Micropores (μg/cm^2^/h)	50 Micropores (μg/cm^2^/h)	100 Micropores (μg/cm^2^/h)
Water	12.56 ± 2.25	41.90 ± 16.56	69.58 ± 17.69	87.55 ± 10.24	98.05 ± 38.15
20% P407	6.77 ± 2.17	17.74 ± 5.53	15.35 ± 4.76	26.94 ± 6.31	24.58 ± 10.39

## Data Availability

The data are available on request from the corresponding author.
